# CIRCADIAN CLOCK-ASSOCIATED1 Delays Flowering by Directly Inhibiting the Transcription of *BcSOC1* in Pak-choi

**DOI:** 10.3390/plants13162190

**Published:** 2024-08-08

**Authors:** Ying He, Dong Xiao, Cheng Jiang, Yiran Li, Xilin Hou

**Affiliations:** 1 National Key Laboratory of Crop Genetics & Germplasm Innovation and Utilization, Key Laboratory of Biology and Genetic Improvement of Horticultural Crops (East China), Ministry of Agriculture and Rural Affairs of China, Engineering Research Center of Germplasm Enhancement and Utilization of Horticultural Crops, Ministry of Education of China, Nanjing Agricultural University, Nanjing 210095, China; 2020204025@stu.njau.edu.cn (Y.H.); dong.xiao@njau.edu.cn (D.X.); 2021204023@stu.njau.edu.cn (C.J.); 2019204021@stu.njau.edu.cn (Y.L.); 2 Nanjing Suman Plasma Engineering Research Institute Co., Ltd., Nanjing 211162, China

**Keywords:** circadian clock, flowering time, *BcCCA1*, *BcFLC*, *BcSOC1*, Pak-choi

## Abstract

Flowering is critical to the success of plant propagation. The MYB family transcription factor *CIRCADIAN CLOCK-ASSOCIATED1* (*CCA1*) is an essential component of the core loop of the circadian clock and plays a crucial role in regulating plant flowering time. In this study, we found that photoperiod affects the expression pattern and expression level of *BcCCA1*, which is delayed flowering time under short-day conditions in Pak-choi [*Brassica campestris* (*syn*. *Brassica rapa*) ssp. *chinensis*]. We detected overexpression and silencing of *BcCCA1* in Pak-choi, resulting in delayed and promoted flowering time, respectively. Furthermore, we also discovered that *FLOWERING LOCUS C* (*BcFLC*) and *SUPPRESSOR OF CONSTANS1* (*BcSOC1*) were expressed significantly differently in *BcCCA1* overexpression and silencing plants compared with control plants. Therefore, we further investigated the interaction relationship between *BcCCA1*, *BcFLC*, and *BcSOC1*, and the results showed that *BcCCA1* and *BcFLC* as a complex interacted with each other. Moreover, both BcCCA1 and BcFLC can directly bind to the promoter of *BcSOC1* and repress its transcription, and BcCCA1 can form a complex with BcFLC to enhance the transcriptional inhibition of *BcSOC1* by BcFLC. This study reveals a new mechanism by which the circadian clock regulates flowering time.

## 1. Introduction

Growth and development in plants are regulated by the circadian cycle, photoperiod, and seasonal temperature changes [1]. Plants have developed a circadian clock regulatory system that integrates internal and external signals to assist plants in adapting more effectively to external changes [2], which is composed of an input pathway, an oscillator, and an output pathway [3]. The circadian clock can produce approximately 24 oscillations in response to environmental changes, influencing the expression of various genes that control metabolic, physiological, and biochemical processes, including activities such as stem growth and flowering induction, and integrating signals from both within and outside the plant to aid in environmental adaptation [4,5].

Flowering serves as a crucial signal for the transition from vegetative to reproductive growth and plays a fundamental role in plant breeding. Plants initiate the flowering process by integrating endogenous signals and responding to changes in the external environment, ultimately ensuring successful reproduction at the right time [6,7]. Botanists have dedicated decades of research to understanding the regulation of flowering time, resulting in the identification of six primary factors that regulate this process: photoperiod [8], temperature [9], gibberellins [10], autonomy [11], vernalization, and age [12,13]. Moreover, recent discoveries suggest that environmental stressors can exert regulatory control over flowering time [14]. Drought conditions induce ABA accumulation in plants, thereby accelerating flowering initiation [15]. Conversely, delayed flowering under salt stress may result from modulation within the photoperiodic pathway involving the *GIGANTEA* (*GI*)-*CONSTANS* (*CO*)-*FLOWERING LOCUS T* (*FT*) module [16].

In plants, *CIRCADIAN CLOCK-ASSOCIATED1* (*CCA1*), a member of the MYB family of proteins primarily functioning as repressors, shows peak expression at dawn and suppresses the expression of *TIMING OF CAB EXPRESSION 1* (*TOC1*) [3,17], while also stimulating the expression of *PRR7* and *PRR9* among pseudo-response-regulated (PRR) proteins [18]. In contrast, these PRR proteins have been shown to inhibit the expression of *CCA1*. Conversely, RVEs encode MYB family transcription factors that counteract the actions of *CCA1* and *LHY* to induce the expression of these same downstream genes [19,20]. Therefore, certain MYB family transcription factors are involved in all major transcription-translation feedback loops constituting the core clock mechanism. As previously mentioned, the circadian clock regulates various physiological activities in plants, including flowering time regulation. In Arabidopsis, cca1 mutant plants exhibit earlier flowering under short-day (SD) conditions; whereas overexpression of CCA1 leads to delayed flowering in Arabidopsis under both long-day (LD) and SD conditions [21]. Previous studies have shown that CCA1 directly binds to the promoters of *EARLY FLOWERING3* (*ELF3*) and *GI* to suppress their expression, leading to delayed flowering in *Arabidopsis* [22]. Furthermore, the MADS box transcription factor *SUPPRESSOR OF CONSTANS1* (*SOC1*) plays a pivotal role as a floral activator, integrating multiple pathways involved in flowering time regulation in *Arabidopsis* [23]. The genes CCA1 and FLOWERING LOCUS C (FLC) have the ability to postpone flowering time by interacting with the *SOC1* promoter and inhibiting its transcription [24]. Additionally, *FLC* could potentially contribute to the natural variation in activity under continuous light conditions, leading to an enhanced late flowering phenotype in the *cca1* mutant [24].

Pak-choi [*Brassica campestris* (*syn*. *Brassica rapa*) ssp. *chinensis*] originates from China and is known for its short growth cycle and nutritional richness [25]. The main edible part of Pak-choi is the leaf; thus, extending the nutritive growth period and delaying the flowering time of Pak-choi have been key breeding objectives for us. Therefore, it is imperative to investigate the mechanism of flowering time regulation in Pak-choi. Previous studies on the regulation mechanism of flowering time in Pak-choi have primarily concentrated on the hormone-regulated pathway for flowering time. For instance, studies have demonstrated that abscisic acid can directly stimulate *BcCO* transcription via *BcABF3*, leading to an acceleration in the transition to flower formation in Pak-choi [26]. Additionally, the expression of the flowering inhibitor gene *B-box 29* (*BcBBX29*) was promoted directly by *ethylene responsive factor 070* (*BcERF070*), which reduced the expression of BcFT and subsequently delayed flowering [27]. Furthermore, earlier studies on the influence of daylength and vernalization on the flowering time of Pak-choi revealed that *BcCCA1* is an environment-specific fQTL related to photoperiodic sensitivity and potentially plays a role in the photoperiodic regulation of flowering time in Pak-choi [28]. Nevertheless, the mechanism by which *BcCCA1* regulates the flowering time of Pak-choi remains unclear.

In this study, we observed a phase delay and significantly higher peak expression of *BcCCA1* under SD conditions compared to LD conditions, resulting in delayed flowering in Pak-choi under SD conditions. Subsequently, we conducted additional investigations into the downstream cascade of flowering time regulators regulated by *BcCCA1*. We discovered that both BcCCA1 and BcFLC have the ability to directly bind to the *BcSOC1* promoter, leading to the repression of its transcription. Moreover, BcCCA1 can interact with BcFLC, forming a complex that enhances the transcriptional repression of *BcSOC1* by BcFLC, thereby playing a regulatory role in the flowering time of Pak-choi. The findings from this study establish a novel foundation and concept for understanding the regulation of flowering time in plants by the circadian clock.

## 2. Results

### 2.1. Identification of BcCCA1

**Figure 1:**
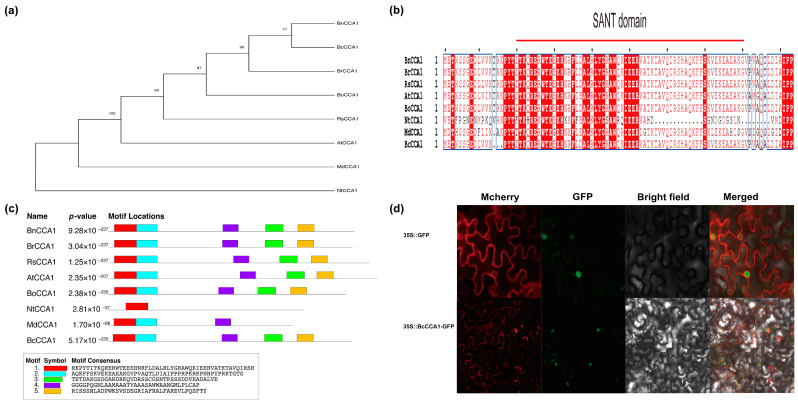
Characterization and subcellular localization of BcCCA1 protein. (**a**) Phylogenetic analysis of CCA1 proteins in different species. (**b**) Multiple sequence alignments of the amino acid sequence of *CCA1*. The SANT domain is highlighted with a red line. (**c**) The motif analysis of *CCA1*. The relevant sequence information is shown at the bottom of the figure, and the *p*-value represents the significance of each motif. (**d**) Subcellular localization of BcCCA1. Plasma membrane markers (PM-mcherry) were used as the membrane markers. Red box with white letter means strict identit. White box with red letter means similarity in a group. Blue frame means Similarity across groups. The pRI 101-GFP vector was used as a control. Scale bars = 50 μm.

Numerous studies have demonstrated that *CCA1*, a MYB family transcription factor, plays a crucial role in regulating flowering time in multiple crops as a core component of the circadian clock genes [21]. To elucidate the sequence structure of *BcCCA1*, we conducted several comparative analyses of *CCA1* amino acid sequences in various horticultural and model plants. Phylogenetic analysis revealed a close relationship between *BcCCA1* and *BnCCA1*, as well as other cruciferous plants (Figure 1a). As depicted in Figure 1b, *CCA1* possesses a conserved SANT domain in all plants except *NtCCA1*, indicating the relatively conserved functions of *CCA1* in most plants. The analysis of conserved domains revealed that *AtCCA1* (*Arabidopsis thaliana*), *BnCCA1* (*Brassica napus*), *BrCCA1* (*Brassica rapa*), *RsCCA1* (*Raphanus sativus*), *BoCCA1* (*Brassica oleracea)*, and *BcCCA1* [*Brassica campestris* (*syn*. *Brassica rapa*) ssp. *chinensis*] exhibited a relatively conserved pattern, each consisting of five identical domains (Figure 1c). The subcellular localization of *BcCCA1* was examined through a transient assay of 35S: BcCCA1-GFP, where the GFP reporter was translated and fused to the 3′ end of *BcCCA1*’s cDNA. The results revealed that BcCCA1 is localized in both the nucleus and the plasma membrane (Figure 1d). In conclusion, *BcCCA1*, a member of the MYB family transcription factors, exhibits structural and functional similarities to *CCA1* found in other plants.

### 2.2. The Expression Patterns of BcCCA1 

**Figure 2:**
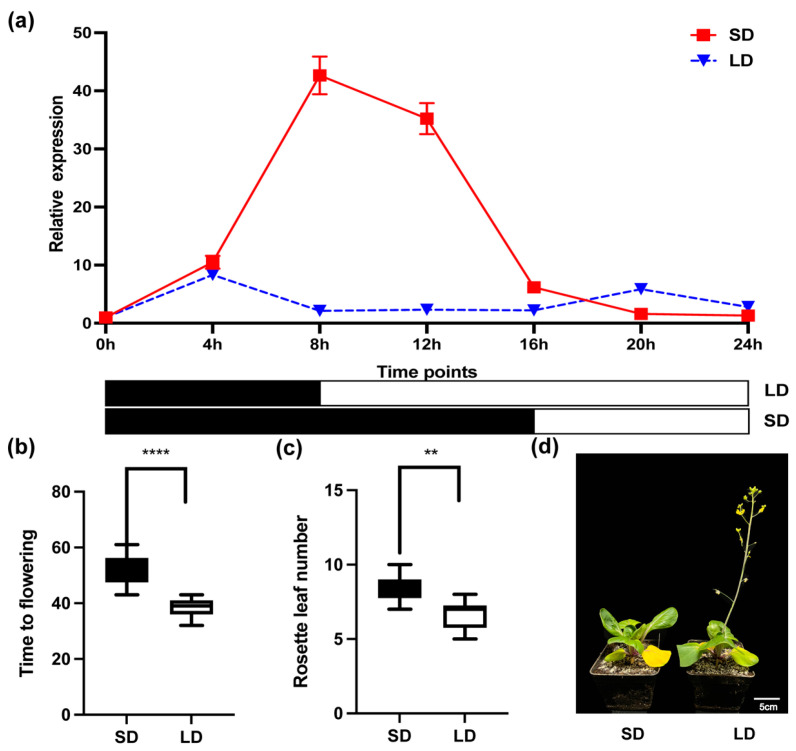
Effects of photoperiod on the expression patterns of *BcCCA1* and flowering time in Pak-choi. (**a**) Expression patterns of *BcCCA1* under LD and SD conditions for 24 h. (**b**) Box plots and whiskers showing the days to flowering of Pak-choi. (**c**) Box plots and whiskers displaying the number of rosette leaves at bolting in Pak-choi. The horizontal line within the box indicates the median value, the boundaries of the box indicate the 25th and 75th percentiles, and the whiskers indicate the highest and lowest values of the results. (Student’s *t*-test, ** *p* < 0.01, **** *p* < 0.0001). (**d**) Photographs of Pak-choi flowering phenotypes under LD and SD conditions.

To investigate the effect of photoperiod on the expression of *BcCCA1* in Pak-choi, we cultivated ‘suzhouqing’ plants under long-day (LD) and short-day (SD) conditions until they reached the flowering stage. Samples were collected every 4 h starting at the age of 20 days, before the plants reached the flowering stage, resulting in a total of 7 sampling points under both LD and SD conditions. Our findings revealed that the peak expression of *BcCCA1* under short-day light was phase-delayed compared to long-day light conditions, and the expression level of *BcCCA1* was significantly higher under short-day light (Figure 2a). Additionally, the SD condition resulted in a delayed flowering time in Pak-choi (Figure 2b–d). In conclusion, our results demonstrate that photoperiod influences the rhythmic expression of *BcCCA1* in Pak-choi and potentially acts as a negative regulator of flowering time.

### 2.3. Analysis of BcCCA1-Overexpressed Plants

**Figure 3:**
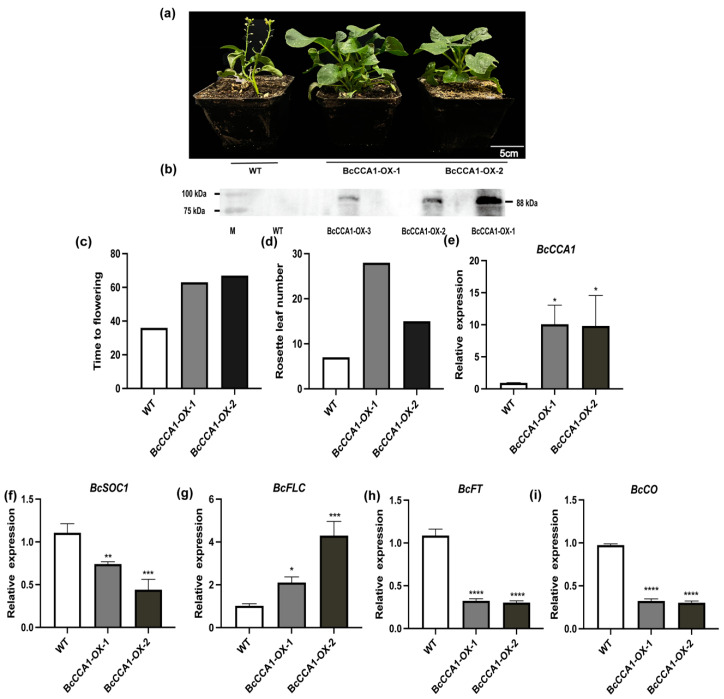
Effect of *BcCCA1* overexpression on flowering time in Pak-choi. (**a**) Phenotypes of plants of WT and *BcCCA1*-overexpressed (*BcCCA1*-OX) plants of ‘Pc-101’. (**b**) The transgenic plants were identified by WB. (**c**) Days to flowering of WT and *BcCCA1*-*OX*. (**d**) Rosette leaf number at bolting of WT and *BcCCA1*-*OX*. (**e**) The expression level of *BcCCA1*. (**f**) The expression level of *BcSOC1*. (**g**) The expression level of *BcFLC*. (**h**) The expression level of *BcFT*. (**i**) The expression level of *BcCO*. (Student’s *t*-test, * *p* < 0.05, ** *p* < 0.01, *** *p* < 0.001, **** *p* < 0.0001).

To explore the role of *BcCCA1* in regulating flowering time in Pak-choi, we generated transgenic plants that overexpressed *BcCCA1* using the ‘Pc-101’ variety. Two transgenic plants were selected for further analysis, and the number of days to flowering and the number of rosette leaves at flowering were recorded (Figure 3a,b). Additionally, we assessed the expression levels of other genes known to be involved in the regulation of flowering. Compared to the control plants, transgenic plants overexpressing *BcCCA1* exhibited a delayed flowering phenotype under LD conditions (Figure 3a,c). Moreover, the number of rosette leaves at flowering was significantly higher in the transgenic plants compared to the control plants (Figure 3d). To elucidate the potential factors contributing to the delayed flowering phenotype observed in *BcCCA1*-overexpressed (*BcCCA1*-OX) plants under LD conditions, we analyzed the relative expression levels of downstream flowering time regulators, including *BcSOC1*, *BcFLC*, *BcFT*, and *BcCO*, as well as *BcCCA1*, in the transgenic plants (Figure 3e–i). As anticipated, the mRNA levels of *BcSOC1*, *BcFT*, and *BcCO* were significantly reduced in the *BcCCA1*-OX plants compared to the control plants (Figure 3f,h,i). Conversely, the mRNA levels of *BcFLC* were notably higher in the *BcCCA1*-OX plants compared to the control group (Figure 3g), which is consistent with the observed delayed flowering phenotype.

### 2.4. Analysis of BcCCA1-Silenced Plants

**Figure 4:**
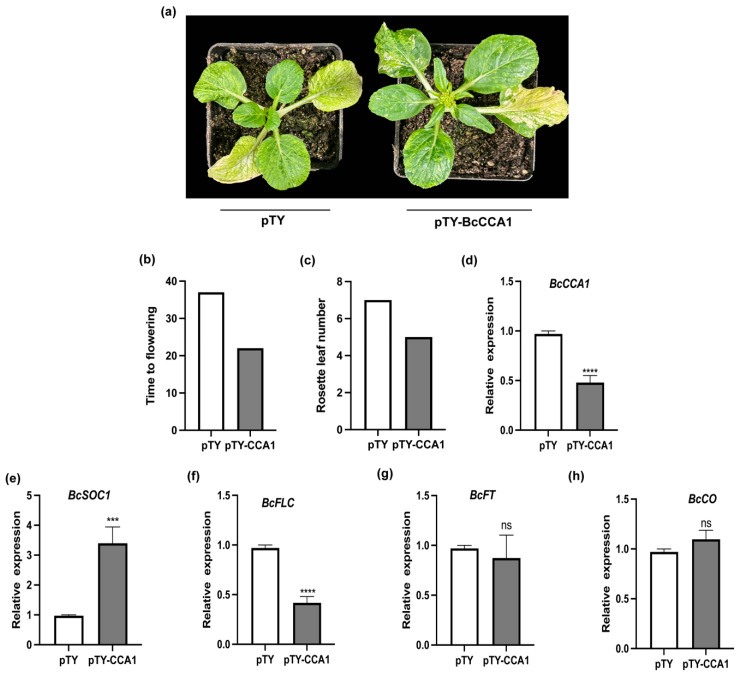
Effect of impaired *BcCCA1* on flowering time in Pak-choi. (**a**) Photographs of the apparently delayed flowering phenotype of *BcCCA1*-silenced. pTY, Pak-choi, injected with an empty vector, was used as the control. (**b**) Days to days to flowering of control and *BcCCA1*-silenced. (**c**) Rosette leaf number at bolting of control and *BcCCA1*-silenced. (**d**) The expression level of *BcCCA1*. (**e**) The expression level of *BcSOC1*. (**f**) The expression level of *BcFLC*. (**g**) The expression level of *BcFT*. (**h**) The expression level of *BcCO*. (Student’s *t*-test, *** *p* < 0.001, **** *p* < 0.0001).

To further investigate the function of *BcCCA1*, we employed virus-induced gene silencing (VIGS) technology to silence *BcCCA1* in Pak-choi plants. The control plants (pTY) were injected with an empty pTY vector as a control (Figure 4a). It was observed that *BcCCA1*-silenced plants exhibited significantly earlier flowering time, compared to the control plants under LD conditions (Figure 4b), and the number of rosette leaves at flowering was lower in the *BcCCA1*-silenced plants compared to the control plants (Figure 4c). The transcript level of *BcCCA1* was reduced in the *BcCCA1*-silenced plants compared to the control plants (Figure 4d). Analysis of the expression levels of *BcSOC1*, *BcFLC*, *BcFT*, and *BcCO* (Figure 4e–h) revealed that there was no significant difference in the expression levels of *BcFT* and *BcCO* between the *BcCCA1*-silenced plants and the control plants (Figure 4g,h). However, the expression of *BcSOC1* was significantly higher in the *BcCCA1*-silenced plants compared to the control plants, whereas the expression level of *BcFLC* was significantly lower in the *BcCCA1*-silenced plants (Figure 4e,f).

### 2.5. The BcCCA1-BcFLC Complex Can Repress the Expression of BcSOC1

**Figure 5:**
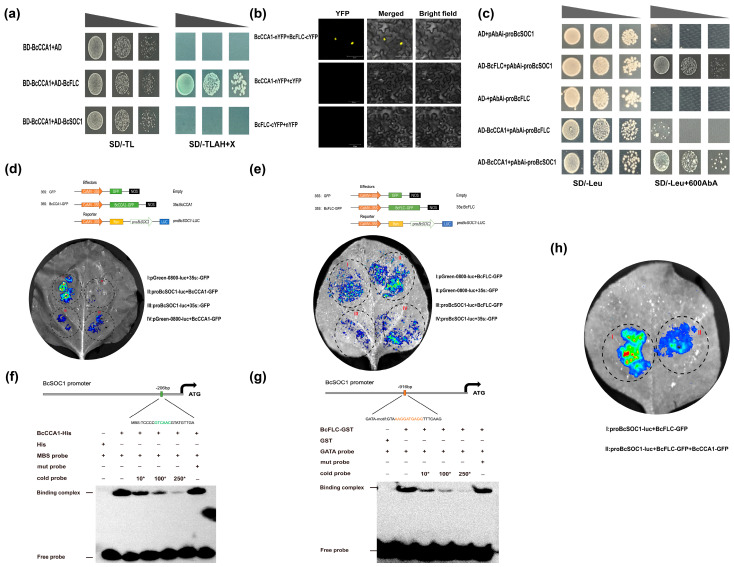
*BcCCA1* and BcFLC repress the expression of *BcSOC1*. (**a**) Interaction between BcCCA1 and BcFLC in the Y2H assay. (**b**) Interaction between BcCCA1 and BcFLC by BiFC assay in tobacco cells. Scale bar, 50 μm. (**c**) The Y1H assay indicated BcCCA1 and BcFLC interact with the promoter of *BcSOC1*. (**d**) Dual-luciferase assay demonstrated BcFLC suppressed the transcription of *BcSOC1*. (**e**) Dual-luciferase assay demonstrated BcCCA1 could suppress the transcription of *BcSOC1*. The EMSA assay indicated BcFLC can bind to the GATA-motif in the promoter of *BcSOC1*. (**f**) The EMSA assay indicated BcCCA1 could bind to the MBS in the promoter of *BcSOC1*. The EMSA assay indicated BcFLC can bind to the GATA-motif in the promoter of *BcSOC1*. (**g**) The EMSA assay indicated BcFLC can bind to the GATA-motif in the promoter of *BcSOC1*. (**h**) The dual-luciferase assay showed that BcCCA1 can enhance the transcriptional repression of *BcSOC1* by complex with BcFLC. 10*, 100* and 250* represent competition between cold probes and biotin probes.

The data presented above indicate that the expression of downstream flowering time regulators is affected by both overexpression and silencing of *BcCCA1*, consequently influencing flowering time in Pak-choi. Notably, *BcCCA1* had a significant impact on the expression levels of *BcFLC* and *BcSOC1*. These observations suggest a correlation between *BcCCA1* and *BcFLC*. To confirm this interaction, we conducted yeast two-hybrid (Y2H) and yeast one-hybrid (Y1H) experiments. The Y2H experiment revealed an interaction between BcCCA1 and BcFLC, whereas no interaction was detected between BcCCA1 and BcSOC1 (Figure 5a). To further validate the interaction between BcCCA1 and BcFLC, a BiFC experiment was conducted. Yellow fluorescent signals were detected in the nucleus of tobacco cells transformed with BcCCA1-nYFP and BcFLC-cYFP constructs. In contrast, no fluorescent signals were observed in the nuclei of tobacco cells co-transformed with either nYFP + BcFLC-cYFP or *BcCCA1*-nYFP + cYFP (Figure 5b). Thus, these findings suggest that BcCCA1 interacts with BcFLC both in vivo and in vitro, potentially regulating the downstream flowering pathway in Pak-choi. The Y1H assay results demonstrated that both BcCCA1 and BcFLC can directly bind to the promoter of *BcSOC1* (Figure 5c). Furthermore, the dual-luciferase assay confirmed that BcCCA1 and BcFLC directly bind to the promoter of *BcSOC1*, leading to transcriptional repression (Figure 5d,e). To gain further insights into the interaction between transcription factors and promoters, an analysis of the *BcSOC1* promoter sequence was conducted, and putative binding sites for BcCCA1 and BcFLC were predicted. Electrophoretic mobility shift experiments revealed that BcCCA1 and BcFLC can directly bind to the *BcSOC1* promoter through the MYB binding site (MBS) and GATA-motif, respectively, in vitro (Figure 5f,g).

Based on the aforementioned results, it is hypothesized that *BcCCA1* may regulate the flowering time of Pak-choi through the *BcFLC*-*BcSOC1* pathway. The dual-luciferase assay was utilized to investigate the impact of the BcCCA1-BcFLC complex on the transcription of *BcSOC1*. The results demonstrated that the fluorescence intensity of 35S:BcCCA1-GFP, 35S: BcFLC-GFP, and pro*BcSOC1*-luc was significantly lower compared to the combination of 35S:BcFLC-GFP + pro*BcSOC1*-luc upon co-injection. This confirms the capability of BcCCA1 to enhance the transcriptional repression of *BcSOC1* by BcFLC (Figure 5h). To further investigate the conservation of *CCA1* function in Pak-choi, we verified its interaction with other clock genes. Experimental results demonstrated that BcCCA1 binds the promoters of *BcTOC1*, *BcELF3*, and *BcGI* in Pak-choi, resulting in the negative regulation of their expression levels. These findings suggest that these interactions are conserved in Pak-choi (Figure S1). In summary, *BcCCA1* regulates flowering time through direct inhibition of *BcSOC1* transcription, in addition to its role in the *CO*-*FT* pathway. Furthermore, the complex formed between BcCCA1 and BcFLC enhances the transcriptional inhibition of *BcSOC1* by BcFLC, thereby delaying flowering time.

## 3. Discussion

Flowering time is a crucial plant trait that significantly influences reproductive success in various external environments. Plants have developed intricate molecular regulatory networks to optimize reproductive efficiency, ensuring flowering occurs in suitable environments [8]. Among these pathways, photoperiod serves as a significant regulatory mechanism for flowering time, which can be classified into three main types based on the plant’s response to changes in day length: LD, SD, and DN [29]. The circadian clock, in response to photoperiodic changes, coordinates the expression of downstream flowering integration genes (e.g., *FT*, *CO*, *FLC*, and *SOC1*) to achieve accurate regulation of plant flowering time [30]. *CCA1*, a widely recognized central circadian clock regulator, plays a crucial role in coordinating plant responses to environmental changes [31]. This study focuses on investigating the temporal expression of *BcCCA1* under LD and SD conditions over a 24-h period. We observed that the peak expression of *BcCCA1* occurred one phase later under SD conditions compared to LD, with a significantly higher intensity (Figure 2a). Additionally, we observed a delay in the flowering time of Pak-choi under SD conditions (Figure 2b–d). In conclusion, the varying durations of light within a light/dark period impact the rhythmic expression of the core circadian clock gene *BcCCA1*, consequently influencing flowering time.

Circadian clocks integrate both circadian and seasonal environmental signals to regulate a wide range of physiological activities, enhancing plant adaptability and competitiveness [32]. *CCA1*, the first circadian clock gene to be cloned in *Arabidopsis thaliana*, plays pivotal roles in regulating various functions, including flowering time, circadian rhythms, leaf movement, hypocotyl elongation, and sensing low temperature environments, among others [22,33,34]. Overexpression of *CCA1* leads to disrupted circadian rhythms and delayed flowering, while *cca1* mutants display shorter rhythmic cycles and early flowering in *Arabidopsis* [33]. Homologous genes of cca1 have also been identified in safflower (*Carthamus tinctorius* L.), *mung bean*, and other species, exhibiting similar expression patterns and conserved functions [35,36]. It is involved in regulating photoperiodic flowering through modulation of the *GI*-*CO*-*FT* pathway [37]. Numerous studies have demonstrated that *CCA1* and *LHY* play a redundant role as negative regulators of *Arabidopsis thaliana* flowering [38]. The expression levels of *BcFT* and *BcCO* in *BcCCA1*-silenced plants were not found to be significantly higher than those in control plants. Therefore, it is speculated that *BcLHY* may continue to negatively regulate the expression levels of *BcCO* and *BcFT* in *BcCCA1*-silenced plants. Subsequent studies will further explore the joint regulatory role of *BcLHY* and *BcCCA1* in flowering regulation, aiming to elucidate the potential mechanism of circadian clock regulation of Pak-choi flower development. In rice, the *LHY*/*CCA1* complex regulates starch metabolism by regulating carbon allocation and starch degradation [39]. The expression levels of *BcSOC1* and *BcFLC* were significantly altered in *BcCCA1*-overexpressing plants and *BcCCA1*-silenced plants compared to control plants (Figure 3a,c and Figure 4a,b). The expression levels of *BcSOC1* and *BcFLC* were significantly different in *BcCCA1*-overexpressing plants and *BcCCA1*-silenced plants compared with control plants (Figure 3f,g and Figure 4e,f). Therefore, we hypothesized that *BcCCA1* may regulate flowering time through the modulation of *BcFLC* and *BcSOC1* expression.

**Figure 6:**
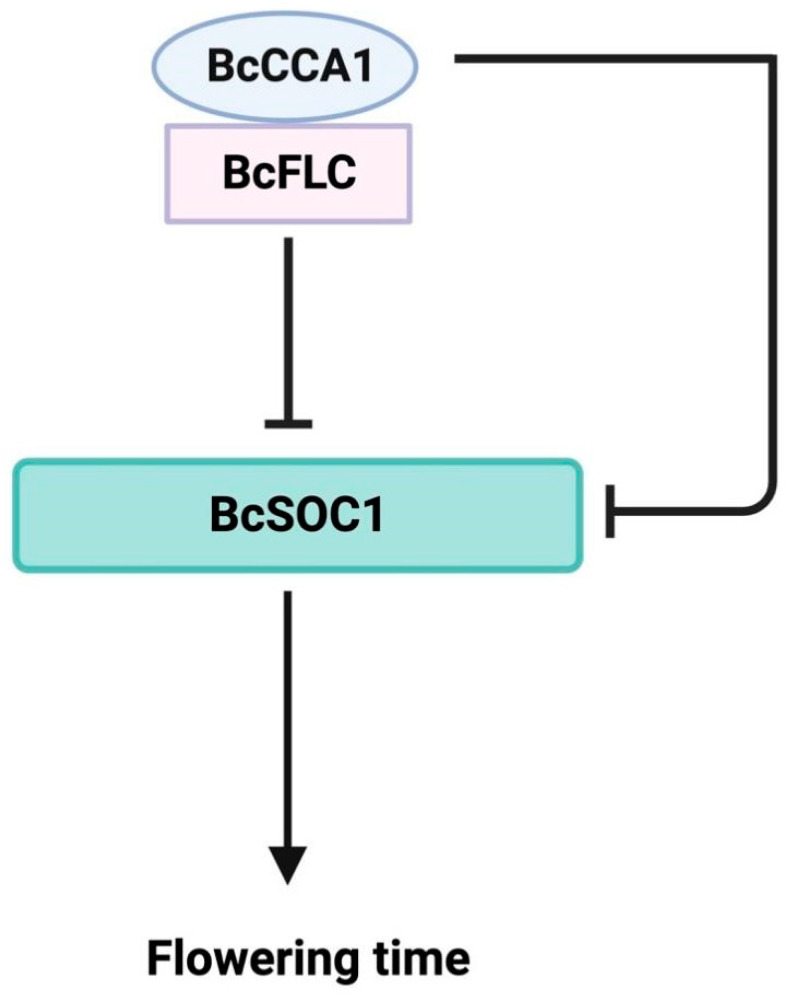
Regulatory pathway of BcCCA1-BcFLC-BcSOC1 regulates flowering time of Pak-choi.

*FLC* is a major effector mediating the vernalization response [40], which delays flowering time by preventing the expression of flower-activating factors [41]. Vernalization can inhibit *FLC* and promote flowering [41]. It has previously been shown that *CCA1* appears to be a major circadian regulator during cold periods and was involved in vernalization and VIN3 transcriptional activation in early winter [42]. In this study, we hypothesized that *BcCCA1* is related to *BcFLC* and *BcSOC1* by comparing the expression of downstream flowering time-regulated genes in *BcCCA1*-OX and *BcCCA1*-silenced plants, and we confirmed the interaction between BcCCA1 and BcFLC both in vitro and in vivo through Y2H and BiFC assays, respectively (Figure 5a,b). Additionally, they may co-regulate the downstream flowering pathway. The Y1H assay and dual-luciferase assay provided evidence that BcCCA1 and BcFLC can directly bind to the promoter of *BcSOC1* (Figure 5c–e), resulting in its transcriptional repression in both in vitro and in vivo settings. The predicted binding sites of BcCCA1 and BcFLC on the *BcSOC1* promoter, as well as the results of the electrophoretic mobility assay, demonstrated that BcCCA1 and BcFLC directly bind the *BcSOC1* promoter through the myb binding sites (MBS) and GATA-motif, respectively. (Figure 5f,g). A dual-luciferase assay was conducted to investigate the effect of the BcCCA1-BcFLC complex on the transcription of *BcSOC1*, confirming that BcCCA1 enhances the transcriptional repression of *BcSOC1* by BcFLC (Figure 5h). In summary, our findings suggest that BcCCA1 inhibits transcription by binding to the *BcSOC1* promoter, in addition to the well-established *CCA1*-*GI*-*CO*-*FT* pathway and the *CCA1*-*ELF3* pathway [43]. Moreover, the formation of a complex between BcCCA1 and BcFLC enhances BcFLC’s inhibition of *BcSOC1*, resulting in a delay in flowering time (Figure 6). 

## 4. Materials and Methods

### 4.1. Plant Materials and Treatments 

Seeds of ‘Suzhouqing’ [*Brassica campestris* (*syn*. *Brassica rapa*) ssp. *chinensis*] and ‘Pc-101’ [*Brassica campestris* L. ssp. *Chinensis var*. *utilis Tsen et Lee*] were generously provided by the Laboratory of Cabbage Systems Biology at Nanjing Agricultural University, Nanjing, China. The ‘Suzhouqing’ plants were cultivated in growth chambers with a light/dark cycle of 16 h/8 h and temperatures of 24 °C/18 °C under LD conditions. For SD conditions, ‘Suzhouqing’ plants were grown with a light/dark cycle of 8 h/16 h and temperatures of 24 °C/18 °C. Leaf samples were collected every 4 h from 7 points on 20-day-old Pak-choi plants, which were not flowering under both long-day (LD) and short-day (SD) conditions. Leaf samples were collected from wild-type, *BcCCA1*-OX plants, and *BcCCA1*-silenced plants following sunrise. For wild-type and *BcCCA1*-OX plants, sampling occurred when wild-type flowering commenced. For control and *BcCCA1*-silenced plants, sampling occurred when *BcCCA1*-silenced plant flowering commenced. Each sample was collected, rapidly frozen in liquid nitrogen, and stored at −80 °C. Three different leaves were independently replicated three times for each time point.

### 4.2. Cloning and Analysis of BcCCA1

The coding sequence (CDS) of the *BcCCA1* gene from the ‘Pc-101’ strain was amplified using the *BcCCA1* primers *BcCCA1-f* and *BcCCA1-r*. The orthologs of *BcCCA1* were identified using the online BLAST tool (https://blast.ncbi.nlm.nih.gov/Blast.cgi, accessed on 10 September 2023). Multiple sequence alignments of homologous proteins were conducted using MEGAX 7.0 software. Conserved motifs were identified and analyzed using the MEME website (http://meme-suite.org/tools/meme, accessed on 13 November 2023). We generated a neighbor-joining phylogenetic tree using MEGAX7.0 with 1000 bootstrap repeats for statistical support. The primers used are listed in Table S1.

### 4.3. Subcellular Localization of BcCCA1 in Tobacco

The stop codon-free CDS of *BcCCA1* was homologously recombined into the plant expression vector pRI101-GFP using the primers listed in Table S1. The recombinant plasmid *BcCCA1-GFP* and the empty plasmid 35S:GFP were separately transformed into the Agrobacterium tumefaciens strain GV3101. The transformed strains were infiltrated into tobacco leaves when the OD_600_ of the bacterial solution reached 0.8–1.0. After 48–60 h, images were captured using a laser scanning confocal microscope (Zeiss, LSM 500, Oberkochen, Germany).

### 4.4. Vector Construction and Transgenic Plant Generation 

A *BcCCA1-GFP* vector was constructed and transformed into an Agrobacterium tumefaciens strain, which was then used for the transformation of ‘Pc-101’ to generate transgenic plants with increased *BcCCA1* expression [44]. The detailed information on primers can be found in Table S1.

### 4.5. Silencing BcCCA1 Expression by the VIGS System

Following the methodology outlined in a previous study [45], the virus-induced gene silencing (VIGS) technique was utilized. An 80 bp palindromic oligonucleotide sequence (ATAACAAAGCAACGTGAAAGATGGACTGAGGAAGAACATATATGTTCTTCCTCAGTCCATCTTTCACGTTGCTTTGTTAT) was designed and inserted into the pTY vector. Following that, 5 μg of plasmid DNA was introduced into 20 mature ‘Pc-101’ leaves using the particle bombardment method. The plants were also inoculated with the pTY vector plasmid to serve as the control group. All plants were grown in a controlled growth chamber, monitored, and sampled for quantitative real-time PCR analysis.

### 4.6. mRNA Extraction and Quantitative Real-Time PCR

Total mRNA was extracted using the RNA Simple Total RNA Kit (Tiangen, Beijing, China). mRNA was reverse transcribed using an Evo M-MLV Mix Kit with gDNA Clean for qPCR (Accurate Biotechnology, Changsha, Hunan, China, AG11728). The real-time RT-PCR analysis was conducted using the Hieff^®^ qPCR SYBR Green Master Mix (Yeasen, Shanghai, China). The relative transcript levels of genes were calculated using the 2^−ΔΔCt^ method. *BcGAPDH* was used as the internal reference gene for analyzing the qRT-PCR data of Pak-choi. The primers used are listed in Table S1.

### 4.7. Yeast Two-Hybrid Assay

Following the established experimental protocol, we conducted yeast two-hybrid (Y2H) assays employing the Matchmaker Gold system (TakaRa, Dalian, China). The full-length CDS of *BcCCA1* was integrated into the pGBKT7 (BD) vector, while the full-length CDS of *BcFLC* and *BcSOC1* were incorporated into the pGADT7 (AD) vector, respectively. Subsequently, these recombinant plasmids were co-transformed into the Y2H GOLD strain. Transformants were cultured on selective media lacking tryptophan and leucine (-Trp-Leu), and monoclonal dilutions were grown on selective media lacking tryptophan, leucine, histidine, and adenine (-Trp-Leu-His-Ade), supplemented with X-alpha-Gal.

### 4.8. Bimolecular Fluorescence Complementation (BiFC) Assay

The stop codon-free CDS of *BcCCA1* and *BcFLC* were fused into the nYFP vector and the cYFP vector, respectively. Subsequently, the recombinant plasmids were transformed into Agrobacterium rhizogenes GV3101 (P19) recipient cells. The combination of the aforementioned bacterial cultures was then injected into tobacco leaves using the transient transformation method. For negative controls, the nYFP + cYFP-BcFLC and nYFP-BcCCA1 + cYFP constructs were subjected to 8 h of dark incubation after injection, followed by 2 d of normal incubation. Observations were carried out using a laser confocal microscope (Zeiss, LSM 780, Jena, Germany).

### 4.9. Yeast One-Hybrid Assay

The 2000 bp sequences upstream of the *BcFLC* and *BcSOC1* transcription start sites were used as promoter regions and fused to the pAbAi vector, respectively. The stop codon-free CDS of *BcCCA1* and *BcFLC* were fused to the pGADT7 (AD) vector. The prey was transformed into Y1H golden yeast strains using the paired golden yeast one-hybrid system from TakaRa and cultured on SD/-Ura medium without AbA (Aureobasidin A) for 3 d. The yeast cells co-transformed with the prey and bait were then cultured in SD/-Leu medium containing 600 ng/mL AbA for 3 d.

### 4.10. Dual-Luciferase Assay

The stop codon-free CDS of *BcCCA1* and *BcFLC* were integrated into the pRI101 vector, and the *BcSOC1* promoter was fused into the pGreen-0800-luc vector. The experiments were conducted following the method described by Hellen [46]. A dual-luciferase reporter gene assay kit (Yeasen) was used to detect luciferase activity after 60–72 h.

### 4.11. Electrophoretic Mobility Shift Assay

The CDS of *BcFLC* and *BcCCA1* were individually cloned into the pGEX-4T-1 (GST) vector and the pcold-His vector, respectively. The resulting recombinant plasmids were then transformed into *Escherichia coli* (DE3)-competent cells. Oligonucleotide probes were synthesized and labeled with biotin at the 5′ and 3′ ends using the online resource (http://www.tsingke.net/). Double-stranded DNA probes were prepared by annealing complementary oligonucleotides. The annealing process involved heating the samples to 95 °C for 3 min, followed by gradual cooling from 1 °C to 25 °C at intervals of 90 s. The resulting probes were then stored at −20 °C. Electrophoretic mobility shift assay (EMSA) detection was performed using a chemiluminescent EMSA kit obtained from (Beyotime, Shanghai, China). Details of the DNA probes used can be found in Table S1.

### 4.12. Statistical Analysis

The experiments were conducted with three circadian replicates and three technical replicates. A student’s *t*-test was employed to analyze the significant differences between each treatment.

## 5. Conclusions

Our study showed that the expression peak value of *BcCCA1* was delayed by one phase under short-day conditions. The expression peak value was also significantly higher under short-day conditions compared to LD. Furthermore, the flowering of Pak-choi was delayed under SD. We further investigated the cascade of flowering time regulators downstream of *BcCCA1*. Our findings revealed that both BcCCA1 and BcFLC directly bind to the promoter of *BcSOC1*, inhibiting its transcription. Furthermore, BcCCA1 interacts with BcFLC to form a complex, which enhances the transcriptional inhibition of BcFLC on *BcSOC1*. This interaction ultimately regulates flowering time in Pak-choi. These findings provide a novel pathway and insights into the circadian clock regulation of plant flowering time.

## Data Availability

Data are contained within the article and Supplementary Materials.

## Supplementary Materials

The following supporting information can be downloaded at: https://www.mdpi.com/article/10.3390/plants13162190/s1, Table S1. Nucleotide sequences of primers used for PCR and RT-qPCR. Figure S1. BcCCA1 repress the expression of *BcELF3*, *BcGI* and *BcTOC1*.
