# Supplementary material for: CIRCADIAN CLOCK-ASSOCIATED1 Delays Flowering by Directly Inhibiting the Transcription of BcSOC1 in Pak-choi
Source: Plants (Basel). 2024 Aug 8;13(16):2190. doi: 10.3390/plants13162190 (PMC11359169; doi:10.3390/plants13162190)
Supplement: Supplementary file 1 [file plants-13-02190-s001.zip › Figure S1.pdf]

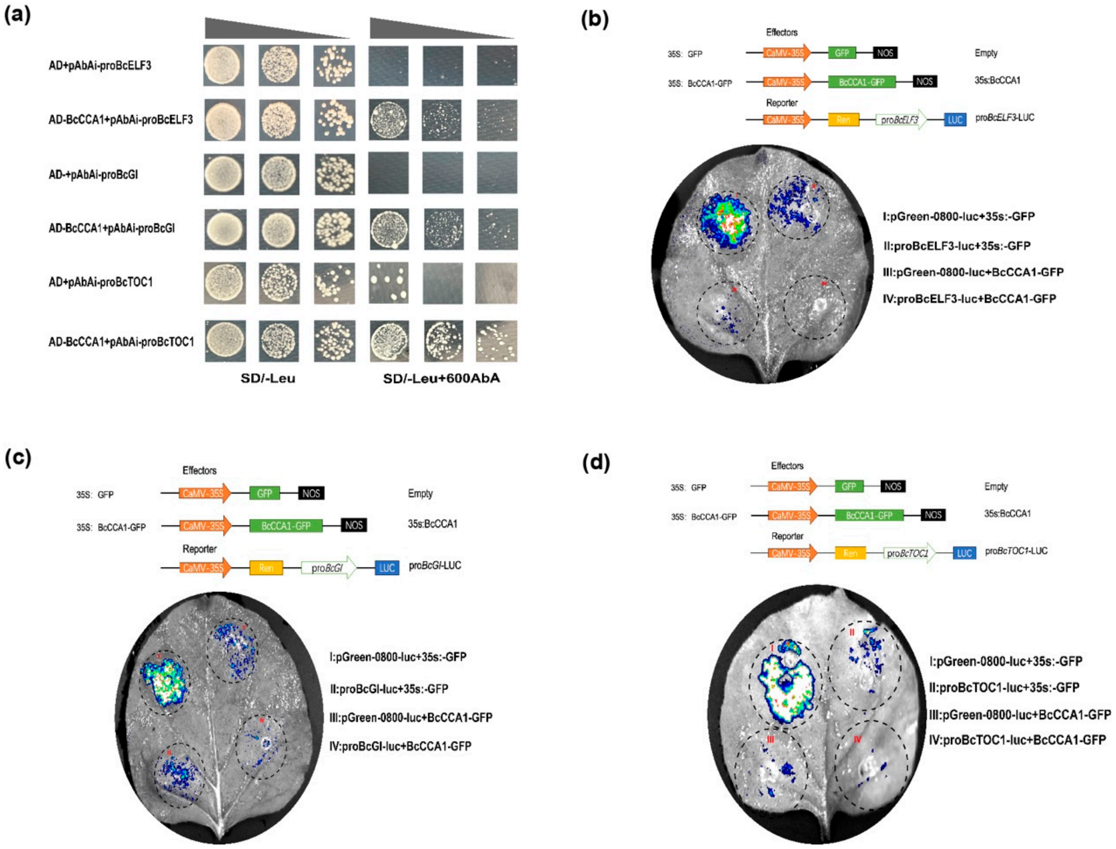

Figure S1. BcCCA1 repress the expression of *BcELF3*, *BcGI* and *BcTOC1*. (a) The Y1H assay indicated BcCCA1 interact with the promoter of *BcELF3*, *BcGI* and *BcTOC1*. (b) Dual-luciferase assay demonstrated BcCCA1 suppress the transcription of *BcELF3*. (c) Dual-luciferase assay demonstrated BcCCA1 suppress the transcription of *BcGI*. (d) Dual-luciferase assay demonstrated BcCCA1 suppress the transcription of *BcTOC1*.
